# Modification of *Salmonella* Typhimurium Motility by the Probiotic Yeast Strain *Saccharomyces boulardii*

**DOI:** 10.1371/journal.pone.0033796

**Published:** 2012-03-19

**Authors:** Rodolphe Pontier-Bres, François Prodon, Patrick Munro, Patrick Rampal, Emmanuel Lemichez, Jean François Peyron, Dorota Czerucka

**Affiliations:** 1 INSERM, U895, Centre Méditerranéen de Médecine Moléculaire (C3M), Equipe Inflammation, Cancer, Cellules Souches Cancéreuses, Nice, France; 2 Université de Nice-Sophia Antipolis, UFR Médecine, IFR50, Faculté de Médecine, Nice, France; 3 Centre Hospitalier Universitaire, Service de Pédiatrie, Hôpital de l'Archet, Nice, France; 4 Centre Hospitalier Universitaire, Service d'Hématologie Clinique, Hôpital de l'Archet, Nice, France; 5 Centre Hospitalier Princesse Grace, Service d'Hépato-Gastro-Entérologie, Monaco; 6 INSERM, U895, Centre Méditerranéen de Médecine Moléculaire (C3M), Equipe Toxines microbiennes dans la relation hôte-pathogènes, France; University of Osnabrueck, Germany

## Abstract

**Background:**

Motility is an important component of *Salmonella enterica* serovar Typhimurium (ST) pathogenesis allowing the bacteria to move into appropriate niches, across the mucus layer and invade the intestinal epithelium. *In vitro*, flagellum-associated motility is closely related to the invasive properties of ST. The probiotic yeast *Saccharomyces boulardii* BIOCODEX (*S.b-*B) is widely prescribed for the prophylaxis and treatment of diarrheal diseases caused by bacteria or antibiotics. In case of *Salmonella* infection, *S.b-*B has been shown to decrease ST invasion of T84 colon cell line. The present study was designed to investigate the impact of *S.b-*B on ST motility.

**Methodology/Principal Findings:**

Experiments were performed on human colonic T84 cells infected by the *Salmonella* strain 1344 alone or in the presence of *S.b-*B. The motility of *Salmonella* was recorded by time-lapse video microscopy. Next, a manual tracking was performed to analyze bacteria dynamics (MTrackJ plugin, NIH image J software). This revealed that the speed of bacterial movement was modified in the presence of *S.b-*B. The median curvilinear velocity (CLV) of *Salmonella* incubated alone with T84 decreased from 43.3 µm/sec to 31.2 µm/sec in the presence of *S.b-*B. Measurement of track linearity (TL) showed similar trends: *S.b-*B decreased by 15% the number of bacteria with linear tract (LT) and increased by 22% the number of bacteria with rotator tract (RT). Correlation between ST motility and invasion was further established by studying a non-motile flagella-deficient ST strain. Indeed this strain that moved with a CLV of 0.5 µm/sec, presented a majority of RT and a significant decrease in invasion properties. Importantly, we show that *S.b*-B modified the motility of the pathogenic strain SL1344 and significantly decreased invasion of T84 cells by this strain.

**Conclusions:**

This study reveals that *S.b-*B modifies *Salmonella's* motility and trajectory which may account for the modification of *Salmonella's* invasion.

## Introduction

A wide range of antibiotics are used to treat human salmonellosis. Moreover, proteomic analysis indicate that all major functions in *Salmonella's* metabolism have already been targeted by antibiotic-based strategies [1]. Moreover, genetic mutation and selective pressure have pushed *Salmonella* spp., as well as other bacteria, to become resistant or multi-resistant to antibiotics [2], [3]. Development of new strategies to prevent or treat infectious diseases has become crucial and targeting of the physical properties of bacteria can constitute such a new strategy.

Since van Leeuwenhoek's first report on bacterial motility in 1683, the majority of bacterial species were found to be motile during at least a part of their life cycle [4]. Movement confers a survival advantage to bacteria by permitting migration towards a favourable microenvironment, or away from an unfavourable one. Movement is closely linked to chemotaxis, the ability to orientate along certain chemical gradients. In case of intestinal pathogenic bacteria, the combination of motility and chemotaxis enables bacteria to detect and pursue nutrients, and to reach their preferred niches for colonization. For instance the intestinal epithelium is covered with mucus glycocalyx, motility likely enables bacteria to pass through and reach enterocytes. In case of enteropathogenic bacteria, motility and chemotaxis have been studied primarily using *Escherichia coli* and *Salmonella enterica* serovar Typhimurim [5], [6].

The flagellum is a bacterial motility apparatus, that in most species can be observed on the cell surface as long filamentous appendices present at one pole in “monotrochously” and “lophotrichlously” flagellated bacteria (e.g., *Helicobacter pylori*, *Vibrio* sp.), at both cells poles in “amphitrichously” flagellated bacteria (e;g., spirochetes or *Spirillum* sp.), or all over the cell body for “petrichously flagellated bacteria” (e.g *Escherichia coli* and *Salmonella* sp.). Structural studies revealed that bacterial flagellum is comprised of three basic parts: the filament (helical propeller), the hook (universal joint), and the basal structure (rotary motor) [reviewed in 7], [8].

Flagella generate bacterial movement via rotation of the filaments and most of flagellar motors are reversible rotary machines, able to rotate both clockwise (CW) and counterclockwise (CCW). In case of “monotrichously flagellated bacteria” the CW and CCW rotations of the flagellum correspond respectively to forwards and backwards swimming modes. In case of “petrichous flagellated bacteria” CCW spinning of the motor generates forces, which cause the individual filaments to sweep around the cell and form a single flagellar bundle propelling the bacterium forward in a “ smooth” swimming motion. When the motor spins CW the propulsive flagellar bundle flies apart and moves individually, thus propelling bacterium in a “tumbly swimming” motion. “Petrichously flagellated bacteria” display a swimming pattern in which the “smooth” and “tumble” modes are alternately repeated [5]. Rotational switching completes very quickly, within only 1 ms and can be achieved by mechanical stress, ions gradients and chemotaxis. Electrochemical gradients of H^+^ and Na^+^ generated across the cytoplasmic membrane, drive motors making it true molecular machine (Mot) that convert electrochemical energy into mechanical work [reviewed in 9]. For chemotaxis, environmental gradients of attractants (amino acids, sugars and oligopeptides) and repellents (extremes pH, some metal ions, hydrophobic amino acids) were perceived by methyl-accepting chemotaxis proteins (MCPs). In the excitation phase, conformational changes caused by ligand binding to MCPs are conveyed to the cytoplasmic face of the membrane where they are recognized by an associated “transmitter” complex (CheA-CheW) [reviewed in 10].

In addition to motility, flagella have roles in other microbial process such as adherence to host cells, cell invasion, protein secretion, autoagglutination, and induction of proinflammatory response in host cells [8].

A correlation between *Salmonella enterica* serovar Typhimurium motility and its property to invade host cells has been initially reported by Jones *et al.* [11]. In that study the authors observed that motility facilitates the collision between bacteria and HeLa cells resulting in attachment to the cell that precedes the invasion. Relationship between motility and invasiveness was confirmed by using different types of mutants, which affect either the flagellar apparatus (app), the flagellum (fla), motility (mot), or chemotaxis (che) [12], [13]. The Che- mutants (cheA, cheW, cheR and cheY) that display “smooth” swimming patterns exhibit more invasiveness than the wild type, but the cheB mutant and mot- (flagellated) strains which are “tumbly” swimming bacteria were found to be less invasive. Tomita and Kagenasaki [14] confirmed this observation by demonstrating that a non-motile flagellated *S.* Typhimurium mutant had a reduced capacity to enter macrophages. Finally, Jones *et al.* [13] compared the invasiveness of different mutants *in vitro* and *in vivo*. The authors clearly established that the “smooth” swimming bacteria entered more easily in contact with Hep-2 cells or Peyer's patches in an intestinal loop model.

Lyophilized *Saccharomyces boulardii* BIOCODEX (*S.b-*B) is a probiotic yeast used for the prevention and treatment of a variety of diarrheal diseases. The mechanism by which *S.b-*B exerts its protective effects was mainly studied in case of infectious diarrhea [15]. *S.b-*B was shown to act via diverse means, including proteolytic cleavage of *Clostridium difficile* toxins A and B [16], [17], and interference with bacterial-stimulated cellular signalling pathways implicated in ions secretion (stimulation of adenylate cyclase) or inflammatory response (MAP kinases, NF-κB pathway) [18]–[22]. In case of *Salmonella* infection we recently reported that *S.b-B* increased survival of *S.* Typhimurium-infected mice and prevented bacterial translocation to the spleen and liver [23]. Cellular studies demonstrated that *S.b-*B decreased the ability of *S.* Typhimurium to invade cells *in vitro*. *S.b-*B also modifies the pro-inflammatory response of host cells infected by ST.

The eminent role of motility in bacterial pathogenesis that offers a basis for a novel anti-infective strategy prompted us to investigate *in vitro* the effect of *S.b-*B on *Salmonella* motility. We report, that the collision between yeast and bacteria are responsible for broken trajectories, modification of bacterial motility and decrease of bacterial invasiveness.

## Results

### *S. boulardii* modifies motility parameters of *Salmonella* Typhimurium

**Figure 1:**
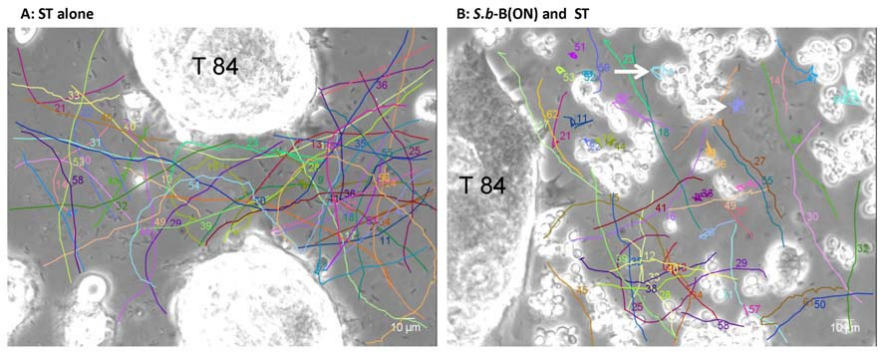
Swimming trajectories of ST incubated alone with T84 cells (A) or cells incubated overnight (ON) with *S.b*-B and exposed to ST (B) derived from a sequence using MTrack J processing software. Records were performed 60 min post infection (PI), and the time between consecutive images was 0.1 second. Using MTrackJ software we determined the locations for each bacterium, and this information was then translated into coordinates (x,y) for each bacterial cell and the process was repeated in times series. The 2D trajectories of each bacterial cell were represented; different colours represent different trajectories. Each trajectory has it own number. Arrows in panel B indicate “rotating” ST trajectories.

Real time computer tracking has been already used to assess the motility of *Rhodobacter sphaeroides*, *Rhodospirullum rubrum* and *Salmonella* Typhimurium [24]. In the present study we used a 2D-time-lapse video microscopy to record bacterial movements in cultures of human colonic T84 cells infected by *Salmonella* Typhimurium alone or in the presence of the probiotic yeast strain *S.b*-B. As tracking of bacterial movements on confluent T84 monolayers was not possible for technical reason (Video S1), records of ST trajectories were made on sub-confluent cells. Records of ST trajectories on cells infected with ST alone or in the presence of *S.b*-B are presented on videos S2 and S3, respectively. As shown on these videos we observed 3 types of bacterial movements: quick with “smooth” swimming trajectories, slow with “tumbly” trajectories, and “rotator” or “spinning” trajectories. Collisions between yeasts and bacteria were also recorded (video S3). Examples of trajectories of bacteria moving in the plane focus and mathematical reconstitution of these ST trajectories are shown in video S4 (Supplementary data) and Figure 1, respectively. A total number of 374 bacteria were manually tracked: 187 bacteria in condition of infection with ST-alone (Fig. 1A) and 187 in condition of T84 cells incubated overnight (ON) with *S.b*-B prior to exposure with ST (Fig. 1B). Strong differences were observed between the trajectory aspects of ST when *S.b*-B was present in the medium (Fig. 1B) and trajectories aspect in ST-alone infected cells (Fig. 1A).

**Figure 2:**
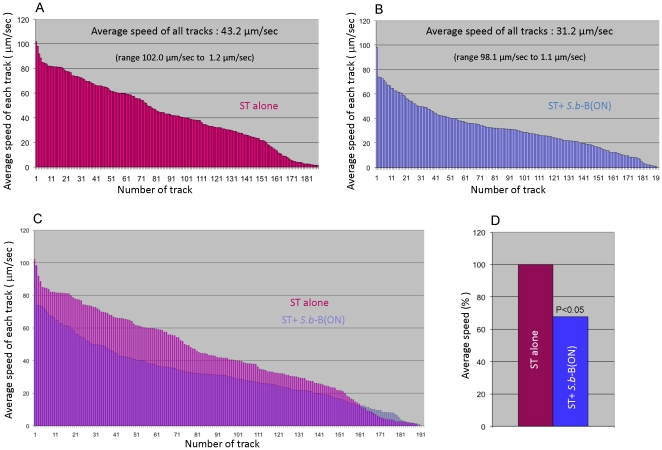
Comparison between the motility of ST alone and ST in the presence of *S.b*-B (ON). The average speeds of 187 ST- alone tracks are presented in panel A and the average speeds of 187 tracks of bacteria in the presence of *S. b*-B (ON) are presented in panel B. Tracks are presented in descending order implying that track number in “x” axis did not correspond to the track number attributed by Image J. Panel C represented the superposition of all tracks from panel A and B. Panel D presented the statistical comparison between the average speeds of ST alone versus the average speeds of ST in the presence of *S. b*-B (ON). The data derived from a sequence using MTrack J processing software.

To further study differences of these trajectories we next went to determine the curvilinear velocity (CLV) and track linearity (TL). Figure 2 shows the CLV of each individual tract reported in descending order in ST infected cells (Fig. 2A), as compared to ST infected cells in the presence of *S.b*–B(ON) (Fig. 2B). In ST infected T84 cells, bacteria moved with a median CLV of 43.2 µm/sec ranging from 102.0 µm/sec for quickly moving bacteria to a speed of 1.2 µm/sec for the slowest bacteria (Fig. 2A). In cells incubated overnight with *S.b*-B and next exposed to ST, the median CLV of ST decreased to 31.2 µm/sec ranging from 98.1 µm/sec to 1.1 µm/sec (Fig. 2B). In order to determine whether quick or slow moving bacteria were affected by yeast cells we represented in Figure 2C the superposition of the tracks. This superposition revealed that *S.b*-B mainly decreased the speed of bacteria that move quicker than 20 µm/sec. Moreover, comparison of the average speeds of ST tracks in both conditions showed a statistically significant decrease by 30%, of the speed of bacteria in the presence of *S.b*-B (Fig. 2D). We hypothesized that the collisions between *S.b*-B and ST might affect flagella properties thus leading to a decrease of bacterial motility.

**Figure 3:**
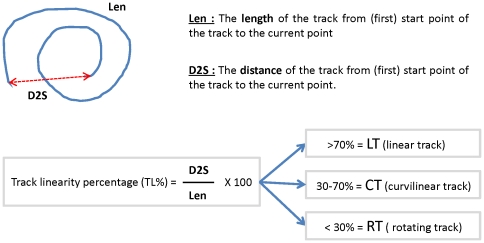
Determination of the linearity of bacterial trajectories.

**Figure 4:**
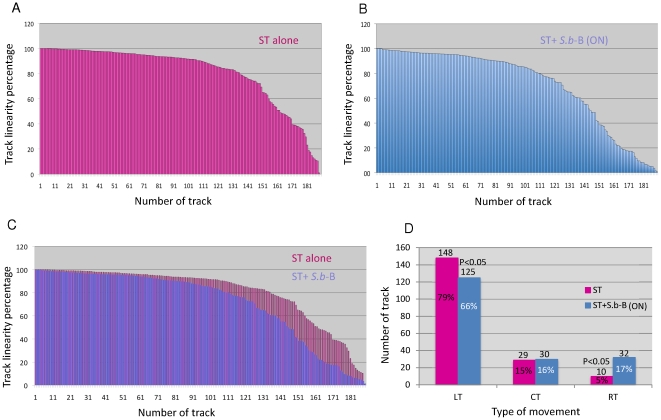
Comparison between the track linearity (LT) of ST incubated alone with T84 (A) or cells incubated overnight (ON) with *S.b*-B (*S.b*-B(ON)) and exposed to ST (B). Tracks are presented in descending order implying that track number in “x” axis did not correspond to the track number attributed by Image-J. 187 ST- alone tracks are presented in panel A and 187 tracks of bacteria in the presence of *S. b*-B(ON) are presented in panel B. In panel C are represented the superposition of the all tracts from panel A and B. Quantification of different types of tracks of ST alone or ST in the presence of *S.b*-B are presented in panel D. The percentages marked in each column correspond to: the number of specific track type/the total number of tracks (187) for each condition ×100. The data derived from a sequence using MTrack J processing software. LT: linear trajectories; CT: curvilinear trajectories, RT: rotatory trajectories.

As presented on Figure 1A and 1B, we observed that microbial collisions also induced modification of trajectory shapes from linear to curve. Mathematically track linearity (TL) can be expressed by the method described by Karim *et al.* [25]. As explained on Figure 3 TL is defined as the ratio between the distance that covers the bacteria “D2S” and the length “Len” of the track. Here we arbitrary defined that when the ratio “D2S”/”Len” is superior to 70% it corresponds to linear track (LT), when this ratio is between 30–70% it corresponds to curvilinear tracks (CT), and finally when it is inferior to 30% it corresponds to rotating tracks (RT). This characterization enables us to identify that CT is unaffected and LT and RT are modified by steric hindrance. Figure 4 shows in descending order the TL percentages measured in cells infected with ST-alone (Fig. 4A) or in the presence of *S.b*-B (ON) (Fig. 4B). In Fig. 4C is presented the superposition of TL from Fig. 4A and B and on Fig. 4D we report the quantification of different types of trajectories. Assessments of track linearity presented in this study showed that the straightness of ST trajectories was modified by *S. b*-B. In ST-alone infected cells 79% of bacteria presented a linear track (LT) that decrease to 66% in the presence of *S.b*-B (ON). Bacteria with rotating movements (RT) were significantly increased from 5% to 17% in the presence of yeast. Bacteria representing a curvilinear track (15% of all tracks in ST- infected cells) were not affected by the presence of *S.b*-B.

### *S. boulardii* affects ST motility by steric hindrance

We next went on studying if the random aspect of collisions could be explained by a difference of size between *S.b*-B and ST. By using the ImageJ plugin we determined the mean yeast and bacteria surfaces reflecting the size of these micro-organisms (see supplementary Figure S1). As depicted in Table 1 the mean size of yeast is 119±55 µm^2^. The important standard deviation is explained by the fact that yeast form population ranging from one isolated cell (mean 40 µm^2)^, to a group containing many cells in case of budding yeast (247 µm^2^). Bacteria are much smaller than yeast with a mean size of 3.4±2.2 µm^2^. Thus our measurements suggest that such big differences in size: 10 times between ST and one cell of *S.b*-B and around 1000 times between one bacteria and a “grape” of yeast cells, might account for high frequencies of collisions.

**Table 1:** Estimation of the surfaces of *S.b*-B, ST and beads.

	Surfaces in µm^2^		
	Means ± SE	Minimal value	Maximal value
*S.b*-B	119±55	40	247
ST (30 min PI)	3.4±0.7	2.2	4.9
beads	29.9±1.8	28.4	32.7

Estimation of the surface of *S.b*-B, ST and beads was made by using ImageJ plugin (more details in supplemental data Figure 1).

**Figure 5:**
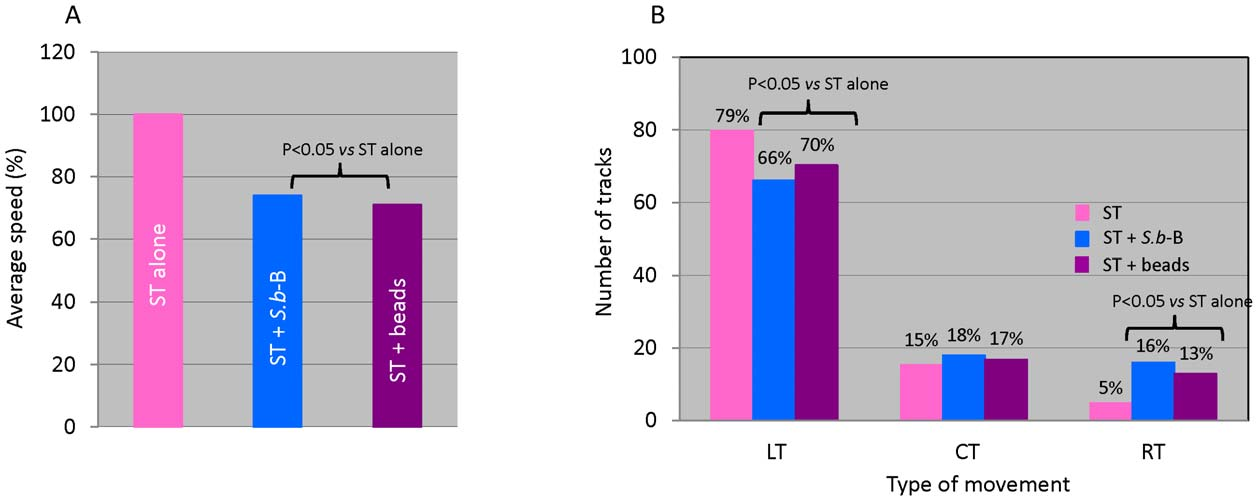
Comparison between motility (panel A) and track linearity (panel B) of ST maintained alone and ST in the presence of *S. b*-B or beads added during infection. Video-microscopic acquisitions were made 30 min PI, and 100 ST were tracked in each condition. In panel A are presented the statistical comparison between the average speeds of ST alone versus the average speeds of ST maintain in the presence of *S. b*-B or beads. In panel B are presented the quantification of different type of tracks: LT, CT or RT. The percentages marked in each column correspond to: the number of specific track type/the total number of track for each condition ×100. The data derived from a sequence using MTrack J processing software.

To further confirm the hypothesis that *S.b*-B may affect ST motility by steric hindrance, we performed experiments in which *S.b*-B was replaced by beads of a similar size. The diameter of beads used in this study was 6.4 µm with a surface calculated of 32.15 µm^2^. As presented in Table 1, the surface of the beads, determined by ImageJ plugin is 29.9±1.8 µm^2^ (see supplementary Figure 1). This value, close to the mathematically defined surface, validates surface determination of micro-organisms by ImageJ. Records of T84 cells exposed during 30 min to ST alone, ST and *S.b*-B or ST and beads are shown on videos S5, S6 and S7, respectively. 100 bacteria were manually tracked for each condition. The average mean speeds and quantification of the different types of trajectories are presented on Figure 5A and 5B, respectively. Addition of beads, as well as addition of *S.b*-B, during infection decreased significantly ST motility by 30% (Fig. 5 A). This showed that the beads had an impact on the distribution of the different types of trajectory (Fig. 5 B). LT trajectories decreased from 79% to 66% in the case of *S.b*-B as compared to 70% with beads. Yeast or beads did not affected CT trajectories. RT trajectories significantly increased in the presence of yeast or beads added during infection. These data clearly demonstrated that the steric hindrance affects both ST motility and trajectory.

**Figure 6:**
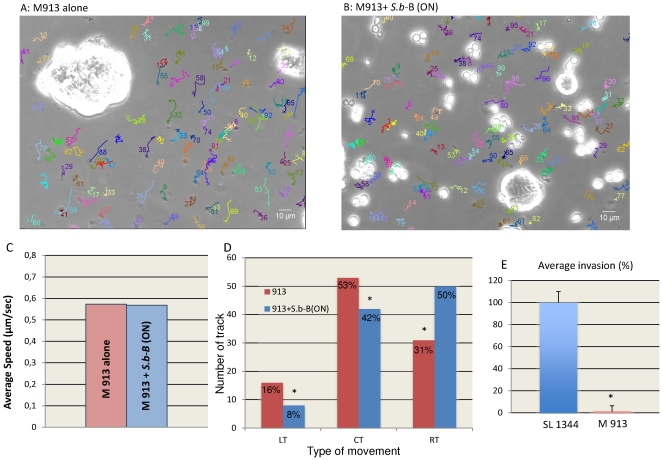
Characteristics of the motility of the mutated strain M913. In panel A and B are presented the swimming trajectories of strain M913 incubated alone with T84 cells (A) or cells incubated overnight (ON) with *S.b*-B and exposed to the strain M913 (B) derived from a sequence using MTrackJ. In panel C are presented the statistical comparisons between the average speed of strain M913 alone versus the average speed of strain M913 in the presence of *S.b*-B(ON). In panel D are presented the quantification of different type of trajectories of strain M913 alone versus the average speed of strain M913 in the presence of *S.b*-B(ON). Data of panels C and D derived from a sequence using MTrack J processing software. In panel E are presented the invasion of T84 cells infected one hour with the strain M913 alone or with the wild type strain SL 1344 alone. Invasion was assessed by the gentamicin protection method [23]. % of invasion was normalized versus SL1344 as 100%. *statistically significant (P<0.05).

### *S. boulardii* decreases ST-invasion of exponentially growing T84 cells

In a previous study we had already demonstrated that *S.b*-B significantly decreased ST invasion of T84 monolayers [23]. As motility assays were performed with exponentially growing cells, we thus investigated if *S.b*-B had also an inhibitory effect on T84 invasion by ST in these new experimental conditions. Data presented in Table 2 show that addition of beads or *S.b*-B during infection significantly decreased invasion of T84 cells by ST. These results established a strong correlation between the modification of the motility of ST by steric hidrance and the decrease of invasive properties of ST. However, in the case of cells exposed overnight to *S.b*-B before infection, invasion was totally abolished (Table 2). This result confirms a previously reported observation made on T84 confluent monolayers [23] showing that overnight incubation with yeast abolished invasion. This result also suggested that both conditioned medium and steric hidrance, together had complementary effects to dampen ST invasion of T84 cells. To verify our hypothesis, we tested whether the conditioned medium (*S.b*-B-CM) also affected ST invasion. Data presented in Table 2 show that *S.b*-B-CM decreased the number of intracellular bacteria to a same extent than after addition of beads. Indeed, we found that to abolish invasion, *S.b*-B-CM must be added together with beads during infection. In parallel, we measured that *S.b*-B-CM also affected ST trajectories (Supplementary Figure S2A and S2C). We also observed that the modification of ST trajectories by *S.b*-CM was more pronounced than in condition of overnight incubation of host cells with *S.b*-B prior to infection (Supplementary Figure S2B). Collectively, our result strongly suggested that in *S.b*-B-overnight incubated cells both steric hindrance and medium composition accounted for dramatic modification of ST motility properties.

**Table 2:** Modification of ST invasion by *S.b*-B, beads or *S.b*-B-CM.

Cell treatment	Intracellular bacteria(×10^4^ CFU/well)Mean ± SEM	% Invasion
SL1344	59.6±12.4	5.9
*S.b*-B+SL 1344	11.0±2.4	1.1*
*S.b*-B (ON)+SL1344	1.8±0.53	0.2*
*S.b*-B-CM+SL1344	11.1±2.85	1.1*
Beads+SL1344	17.2±2.03	1.7*
*S.b*-B-CM+beads+SL1344	5.6±2.56	0.6 #

T84 cells were infected 60 min with the wild type strain SL 1344 alone or in the presence of *S.b*-B, beads or the conditioned medium by the yeast (*S.b*-B-CM) added during the infection. Experiments were also performed in cell incubated overnight (ON) with *S.b*–B (*S.b*-B(ON)) prior infection. At least beads and *S.b*-B-CM were added together during infection with ST. Invasion was assessed by the gentamicin protection method (23). % of invasion was calculated as intracellular bacteria/CFU of ST added by well (10^7^ CFU/well). * Indicates statistical difference *vs* ST-alone infected cells (P<0.05) (n = 4).

# Indicates statistical difference *vs* beads+SL1344 infected cells or *S.b*-B-CM+SL1344 (P<0.05) (n = 3).

### Correlation between ST motility and invasion of T84 cells

Initially, Jones *et al.* [11] reported a correlation between the swimming behavior of ST and its invasiveness properties in HeLa cells. This correlation was never described for human colonic T84 cells. This was first studied with the non-motile, flagella deficient mutant strain M913 [26]. Motility in condition of T84 cells infected by this mutated strain was followed as previously described by video-microscopy. Records presenting exponentially growing T84 cells exposed to the strain M913 alone or in the presence of *S.b*-B are presented on video S8 and S9, respectively. As presented on these videos, strain M913 moved very slowly when compared to the wild type presented in video S2. Reconstitution of track linearity of the strain M913 alone or in the presence of yeast are presented on Figure 6A and B. A total number of 60 bacteria were manually tracked for each condition. Mathematically reconstitution of CLV (Fig. 6C) shows that strain M913 moved with a speed 0.57 µm/sec that is significantly slower than the speed of the wild type (Fig. 2A). The presence of *S.b*-B did not affect the CLV of strain 913 (Fig. 6C). Assessments of track linearity presented on Figure 6D showed that the strain M913 presented only 16% of LT as compared to 79% of LT for the wild type (Fig. 4D). This strain presented mainly CT (53%) and RT (31%) that are significantly higher than the wild type (29% and 10% respectively for CT and RT as presented on Fig. 4D). We found that in case of infection conducted with the strain M913 the presence of *S.b*-B decreased the number of LT and CT to 8% and 42% respectively (Fig. 6D). Conversely, *S.b*-B significantly increased the number of RT to 50% (Fig. 6D).

We next evaluated the number of intracellular bacteria in T84 cells infected by the wild type strain SL1344 or the mutated strain M913. We used the classical gentamycin protective assay [23] to measure the percentage of intracellular bacteria found in infected T84 cells. As presented in Figure 6E the number of intracellular bacteria decreased significantly in the case of the non-motile strain M913 when compared to SL1344. Altogether these data established a correlation between ST motility and invasion.

## Discussion

Real time computer tracking has been already used to assess the motility of *Rhodobacter sphaeroides*, *Rhodospirullum rubrum* and *Salmonella* Typhimurium [24]. In this study we performed video records of T84 cells exposed to ST alone or in the presence of *S.b*-B. Using MTrackJ plugin we performed mathematical reconstitution of trajectories in x, y plane allowing us to determine bacterial velocity (CLV) and linearity (LT). Data reported in this study show that in ST-infected cells, bacteria moved with a median CLV of 43.2 µm/sec ranging from 102.0 µm/sec to 1.2 µm/sec. These values are in great agreement with those reported by other authors for bacteria swimming in fluid [27], [28]. Both values appeared to represent physical limits. For bacteria that moved slower than 1 µm/sec, the value of movement must be lost as the nutrient diffuses faster than the bacterium can find it. The faster speed values must encounter the physical limits of the maximum motor rotation rate and the length and number of flagella. In the case of ST, swimming speed value has been reported as a linear function of the flagellar rotation speed [29]. The rotation rates of the motor varied between 270 r.s^−1^ for *Escherichia coli* and 170 r.s^−1^ for *Salmonella* Typhimurium [30]. Implication of flagella in ST motility was confirmed by the use of the mutated strain M913. These bacteria that were deleted in flagella [26] moved with a median CLV of 0.57 µm/sec that is significantly lower than the CLV of the wild type strain.

Given that cell invasion is a main step in Salmonella pathogenesis, we have investigated invasion in conditions of human colonic T84 cells infected by the wild type strain SL1344 and the non-motile mutated strain 913. We show that invasion of T84 cells by the M913 strain is significantly less efficient (around 100 times) than invasion by the wild type strain. These results established a correlation between ST motility and invasion of the human colonic T84 cells. Initially, Jones *et al*. [11] have reported a correlation between the swimming behavior of ST and its invasive property studying HeLa cells. The authors observed that motility facilitated the contact between bacteria and cells whereupon bacteria became attached in a reversible manner (i.e bacteria could be eliminated by washing of the monolayer). The reversible attachment was a necessary interlude before the bacteria became irreversibly attached to cell. Only irreversibly attached bacteria proceeded to the third phase and were internalized by HeLa cells. In the same study, the direct implication of ST-motility was confirmed by two experiments. In the first one, a parental mobile strain was cultivated in agar medium, which decreased their motility. The second experiment used a non-motile mutated strain. In both cases, bacteria failed to either reversibly or irreversibly attach to HeLa cells and no intracellular bacteria were found. An average of 6 flagella or more were observed for motile bacteria whereas bacteria grown on agar possessed only one. We made similar observation using either HeLa or T84 cells infected with parental SL 1344 strain growing either without shaking (condition that preserves flagella synthesis) or with shaking (condition in which flagella are destroyed). We observed that invasion, as well as the pro-inflammatory response i.e activation of NF-κB nuclear translocation, were significantly reduced in the case of infection by ST cultivated under shaking conditions (data not shown). For that reason in the current study ST were cultivated without shaking to preserve flagellar motility.

Data presented in this study show that the presence of *S.b*-B during the first hour of infection decreased the median CLV of ST to 31.2 µm/sec ranging from 98.1 µm/sec to 1.1 µm/sec. The presence of yeast mainly affected bacteria that moved quicker then 20 µm/sec. In the same time *S.b*-B significantly decreased the number of intracellular bacteria (from 5.9 to 0.2%). This confirmed that a decrease of ST motility is correlated with its invasiveness and can play an important role in the mechanism of action of *S.b*-B.

The key parameters in bacterial movement are absolute speed, constancy of speed, turn angle, gradient length, receptor sensitivity and finally the extent at which the random walk can be biased. Rectification of swimming bacteria by arrays of asymmetric barriers have been studied by Wan *et al.* [31]. This study demonstrated that collision between bacteria and barrier is enough to induce motor force change and modification of bacterial trajectories. These modifications depend on the scale of the barrier. Data presented in our study show that difference in size between ST and one cell of *S.b*-B is 10 time, and around 1000 times between one bacteria and a “grape” of yeast cells. This could explain, in part, the high frequencies of collisions. This may also explain the modification of ST trajectories. ST trajectories that are mainly linear (79%) are decreased to 66% in the presence of yeast. Bacteria presenting rotating trajectories significantly increased from 5% to 17% in the presence of yeast. Since the strain M913 presented also mainly RT trajectories, we can speculate that the increase of the number of rotating bacteria in the presence of *S.b*-B likely account for the decrease of invasion. This observation is confirmed by the data presented with the low invasive strain M913. This flagella mutated strain, that presents 100 times less invasiveness than the wild type SL1344, also presented a lower number of LT trajectories and a significantly higher number of RT trajectories as compared to the wild type strain.

**Figure 7:**
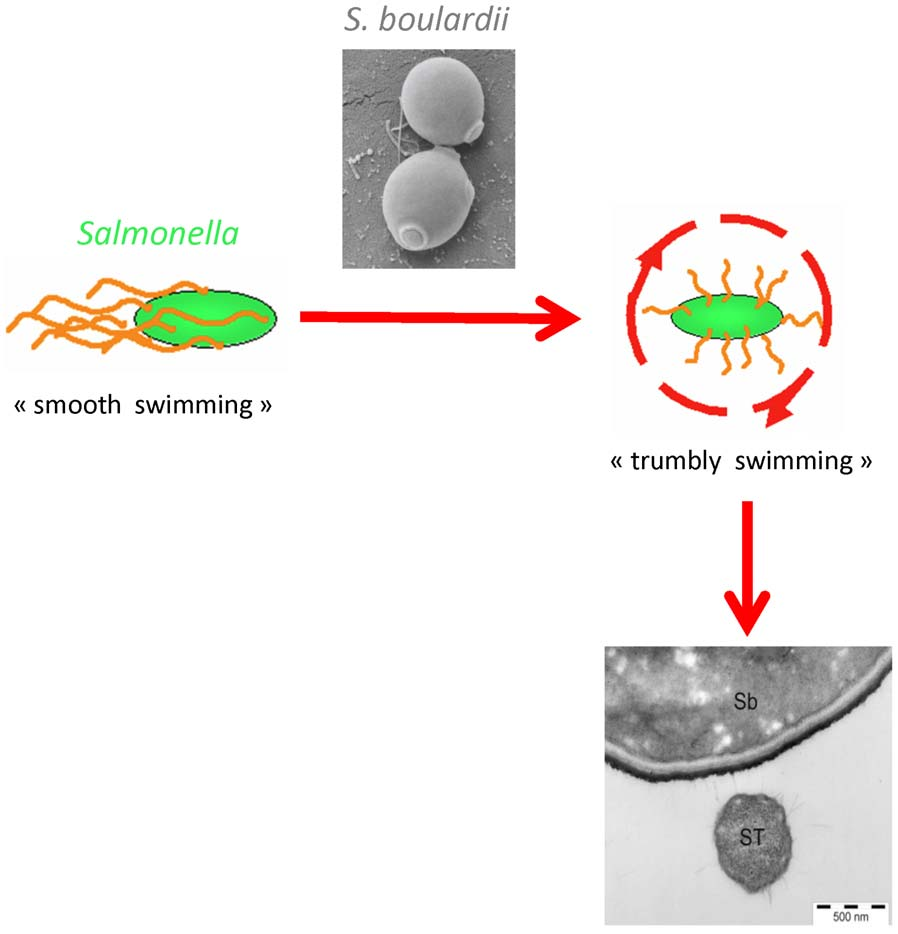
Model of interaction between *S.b*-B and ST. The collision between “smooth” swimming ST and the yeast, induces a change in flagella organisation that modifies ST motility in a ‘tumble” mode, decreasing the speed of bacteria, changing the trajectories and finally enabling the adhesion of ST to yeast as previously observed by electronic microscopy [23].

Altogether these data support the following mechanism of action depicted on Fig. 7. In early phase of infection, collisions between ST and *S.b*-B, modify the motility of ST from “smooth” swimming to a ‘tumble” mode. The collision also modifies the trajectories of bacteria by significantly increasing the number of bacteria with rotator movement. These modifications in motility and trajectories probably enable, in the late phase of infection, the adhesion of ST to yeast as it was previously observed by electron microscopy [23].

Our data show also that *S.b*-CM, decreased the invasion of T84 cells by ST in the same proportion as does the addition of yeast during infection but it did not abolish the invasion as it is the case of cells exposed overnight to *S.b*-B prior to infection. However, when *S.b*-CM and beads are added concomitantly during infection, ST invasion is abolished demonstrating that the yeast acts trough at least two mechanism: one as steric hindrance and the second imply some factors founded in the *S.b*-CM.

## Materials and Methods

### Microorganisms

*Salmonella enterica* serovar Typhimurium (ST) strain SL1344 was kindly provided by Stéphane Méresse, Faculté des Sciences de Luminy, Centre d'Immunologie de Marseille-Luminy (CIML), INSERM-CNRS, Marseille, France. Mutated strain M913 (flagellin -) was kindly provided by Wolf-Dietrich Hardt from Institute of Microbiology, D-BIOL, ETH Zurich, Switzerland. The genetic background and characteristics of these strains are listed in Table 3. Bacteria were stored in Luria-Bertani (LB) medium plus 15% glycerol at −80°C and grown in LB broth overnight at 37°C without shaking. Cultures of *S. boulardii* (*S.b*-B) were obtained by inoculating a commercial lyophilized preparation of the yeast (Ultra-Levure®, BIOCODEX, France) and growing overnight at 37°C, with shaking, in Halvorston minimal medium with 2% glucose.

**Table 3:** Strains used in this study.

Strain	*Relevant Genotype*	Characteristics	Swimming (microscopy)	Reference
SL1344	*Wild type*	Mot+, flag+, che+	+	34
M913	*fli*GHI::Tn10	Mot−, flag−, che+	−	26

### Cell lines and growth conditions

The human T84 colonic cell line was obtained from the European Collection of Animal Cell Cultures (Salisbury, England). The T84 culture medium contained a 1∶1 mixture of Dulbecco-Vogt modified Eagle medium and Ham's-F12 medium (DMEM/F12) supplemented with 50 µg ml^−1^ penicillin, 50 µg ml^−1^ streptomycin (Sigma, France), and 4% fetal bovine serum (Hyclone, France).

### Infection procedure for video-microscopy

T84 cells were seeded at a density of 10^6^ cells/dishes in a 35 mm glass bottom dish (Mat Tek Corporation, USA). 24 hours later culture medium was changed to medium without serum nor antibiotics for 12 hours. Infection was performed with wild type strain SL1344 (ST) or mutated strains M913. Before infection, these strains were grown overnight into LB broth medium without shaking (condition that preserved flagellum). Bacteria were pelleted by gentle centrifugation (2500 rpms for 5 min), re-suspended in DMEM/F12 medium, and added to cells (10^7^ bacteria dishes^−1^). When infection was performed in the presence of *S.b*-B, 10^6^ yeasts were added by dishes overnight before infection (*S.b*-B-ON) or added during infection (*S.b*-B) as determined in different set of experiments. When infections were performed in the presence of beads, beads of 6.4 µm of diameter (Sigma, France) were added during infection. For time-lapse video microscopy, Petri dishes containing infected samples were placed in a humidity (95%), CO2 (5%) and temperature (37°C) –controlled environment for 30 or 60 min as determined for each set of experiments.

### Records of video-microscopy

Motile bacteria and cells were recorded by phase-contrast microscopy using a Leica DMI6000 B inverted microscope equipped with a high- sensitive Ropper CoolSnap HQ^2^ CCD camera (Photometrics) at 40× magnification (numerical aperture: 0.75, Leica HCX PL Fluotar PH2). The optimal time interval between each acquisition was determined for each video acquisition and was below 0.1 seconds, and images were acquired with the LAS-AF software (Leica, Germany).

### Analysis of data from video microscopy

Video sequences were analyzed using the plugin MTrackJ (Image J, NIH, USA) that allows a manual tracking of individual bacteria trajectories (for details see video 4 in Supplementary data). Analyses were only made for bacteria moving in the plane focus in the regions of Interest (ROI), meaning that in all experimental conditions, analysis start and stop when the bacteria appeared and disappeared from the x, y plane. Such analysis allowed mathematical reconstitution of bacterial trajectories in x, y planes.

MTrackJ also allows the determination of curvilinear velocity (CLV) that reflects bacterial speed and of the linearity of the tracks (TL) (see: www.imagescience.org/meijering/software/mtrackJ).

CLV corresponds to the full length of the track divided by its duration. The full length of the track is determined from the first point to the last point of the track. CLV is measured in mm/sec and reflects bacterial speed.

TL is the ratio of distance covered by the bacteria defined as “D2S” column in M Track to the “Len” of the track (“D2S”/”Len”) and is expressed as a percentage (Fig. 3). For a bacterium that runs straight this value is 100%. For a bacterium that spins around a point the “D2S” may be so small compared with the “Len” that the value may approach 0%. Thus, depending of the straightness or curvature of the path of the bacterium the value of track linearity will be between 100% and 0%.

### Infection procedure for invasion assays

T84 cells were seeded into six-well tissue culture plates at 10^6^ cells per well. 24 hours later, culture medium was changed to medium without serum and antibiotics and maintained in this medium overnight. Infection was performed as described below for video microscopic procedure. Bacterial adhesion to T84 cells was quantified using the plate dilution method as previously described [23]. After 1 h of infection, bacteria and yeasts or beads were eliminated by extensive washes with sterile PBS. Cells were then incubated for an additional hour with DMEM/F-12 containing 100 µg of gentamicin per ml. Since gentamicin was not concentrated in epithelial cells, intracellular bacteria survived to the incubation, while adherent and extracellular bacteria were killed. The monolayers were then washed with sterile PBS, and epithelial cells with intracellular bacteria were detached by trypsin and lysed as described elsewhere. Different dilutions of the suspension were plated on LB-agar medium for colony forming unity (CFU) number determination.

### Statistical analysis

All the experiments were repeated at least three times. Results are presented as the mean ± the standard error of the mean (SEM). Statistical significance was determined by analysis of variance with the StatView program for MacIntosh, followed by post hoc comparison with the Bonferroni and Dunn tests. The level of significance was set at *P*<0.05. Analysis of *Salmonella* invasion was performed by Student's *t* test with a P value of <0.05 being considered significant.

## Supporting Information

Video S1 **Movement of bacteria in ST-alone infected T84 monolayers.** Records were performed 60 min PI.
(AVI)

Video S2 **Movement of bacteria in ST-alone exponentially growing -T84 cells.** Records were performed 60 min PI.
(AVI)

Video S3 **Movement of bacteria in exponentially growing -T84 cells incubated overnight with** ***S.b*** **-B and exposed 60 min to ST.**
(AVI)

Video S4 **Example of manual tracking using MTrack J plugin.**
(AVI)

Video S5 **Movement of bacteria in ST-alone exponentially growing -T84 cells.** Records were performed 30 min PI.
(AVI)

Video S6 **Movement of bacteria in exponentially growing -T84 cells exposed for 30 min to ST and** ***S.b*** **-B.**
(AVI)

Video S7 **Movement of bacteria in exponentially growing -T84 cells exposed for 30 min to ST and beads.**
(AVI)

Video S8 **Movement of bacteria in exponentially growing -T84 cells exposed for 60 min to strain M913.**
(AVI)

Video S9 **Movement of bacteria in exponentially growing -T84 cells incubated overnight (ON) with** ***S.b*** **-B and exposed for 60 min to strain M913.**
(AVI)

Figure S1 **Shows the measurement of bacterial, yeast and beads surfaces using ImageJ plugin.**
(XLS)

Figure S2 **Swimming trajectories of ST incubated alone with T84 cells (A), cells incubated overnight with** ***S.b*** **-B before infection (panel B) and with** ***S.b*** **-B -conditioned medium (** ***S.b*** **-B-CM) during infection (panel C).** *S.b*-B-CM was prepared after overnight incubation of yeast in cell culture medium without serum or antibiotics. Yeast were eliminated by centrifugation and T84 cells were incubated with *S.b*-B-CM and ST. Records were performed 60 min PI, bacterial trajectories were determined using MTrackJ software as described above.
(PPTX)
