# Supplementary figures and images for: Modification of Salmonella Typhimurium Motility by the Probiotic Yeast Strain Saccharomyces boulardii
Source: PLoS One. 2012 Mar 19;7(3):e33796. doi: 10.1371/journal.pone.0033796 (PMC3307767; doi:10.1371/journal.pone.0033796)

## Slide 1
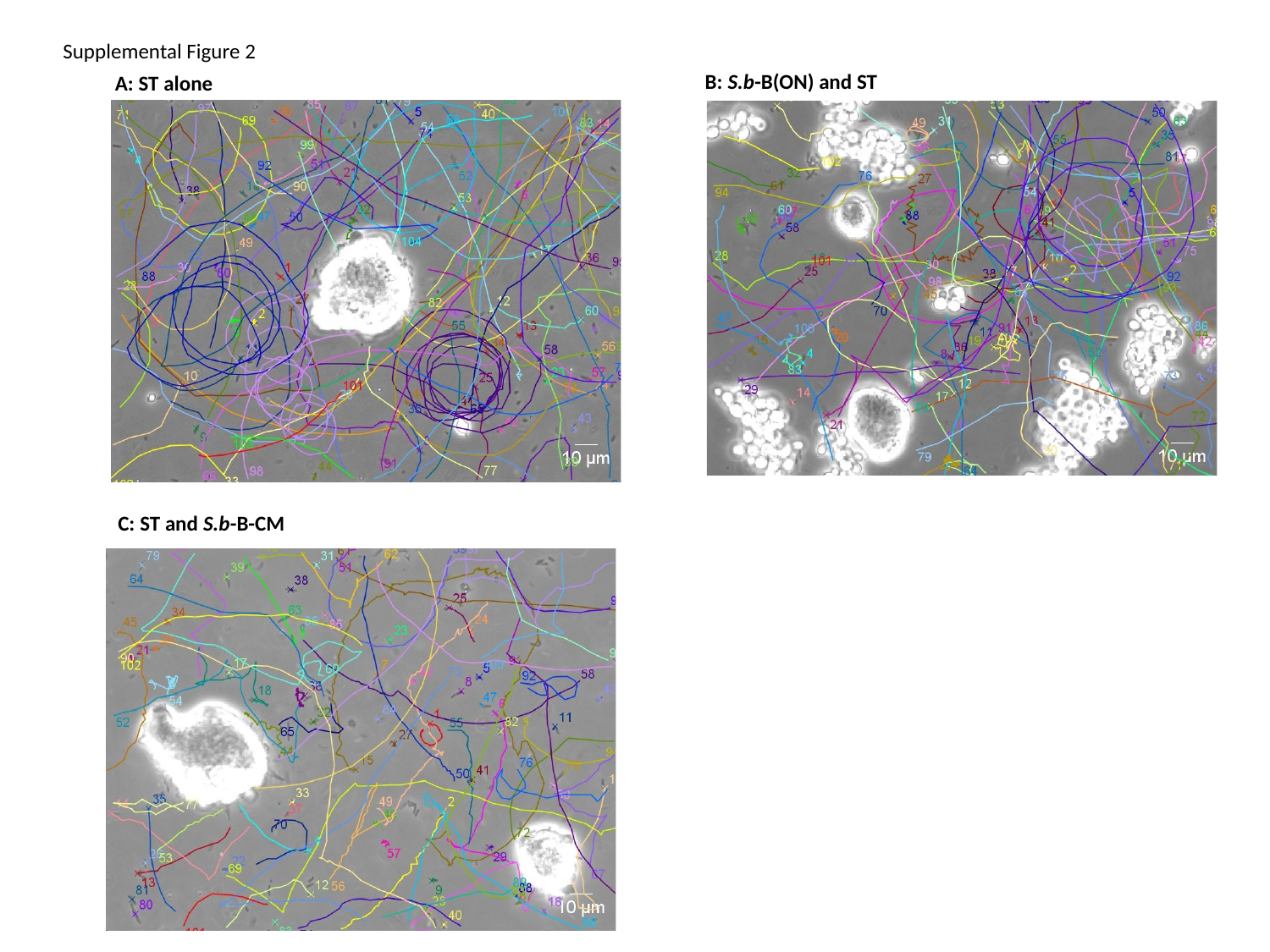

Supplemental Figure 2
B: S.b-B(ON) and ST
 A: ST alone
C: ST and S.b-B-CM

Supplement: Figure S2 — Swimming trajectories of ST incubated alone with T84 cells (A), cells incubated overnight with S.b -B before infection (panel B) and with S.b -B -conditioned medium ( S.b -B-CM) during infection (panel C). S.b-B-CM was prepared after overnight incubation of yeast in cell culture medium without serum or antibiotics. Yeast were eliminated by centrifugation and T84 cells were incubated with S.b-B-CM and ST. Records were performed 60 min PI, bacterial trajectories were determined using MTrackJ software as described above. (PPTX) [file pone.0033796.s011.pptx]
